# Selective inhibition of canonical STAT3 signaling suppresses K-ras mutant lung tumorigenesis and reinvigorates anti-tumor immunity

**DOI:** 10.3389/fimmu.2025.1575181

**Published:** 2025-04-28

**Authors:** Michael J. Clowers, Zahraa Rahal, Sung-Nam Cho, Avantika Krishna, Bo Yuan, Leticia G. Hamana Zorrilla, T. Kris Eckols, Moses M. Kasembeli, Samuel Liu, Stephen Peng, Marco Ramos-Castaneda, Annamarie L. Thompson, Carlos Ignacio Rodriguez Reyna, Katherine E. Larsen, Maria T. Grimaldo, Shanshan Deng, Nastaran Karimi, Cody Chou, Walter V. Velasco, Melody Zarghooni, Sayan Alekseev, Luisa M. Solis Soto, Edwin J. Ostrin, Humam Kadara, Suhendan Ekmekcioglu, David J. Tweardy, Seyed Javad Moghaddam

**Affiliations:** ^1^ Department of Pulmonary Medicine, The University of Texas MD Anderson Cancer Center, Houston, TX, United States; ^2^ UTHealth Houston Graduate School of Biomedical Sciences, The University of Texas M.D. Anderson Cancer Center, Houston, TX, United States; ^3^ Department of Translational Molecular Pathology, The University of Texas MD Anderson Cancer Center, Houston, TX, United States; ^4^ Department of Melanoma Medical Oncology, The University of Texas MD Anderson Cancer Center, Houston, TX, United States; ^5^ Department of Infectious Diseases, Infection Control & Employee Health, The University of Texas MD Anderson Cancer Center, Houston, TX, United States; ^6^ Department of General Internal Medicine, The University of Texas MD Anderson Cancer Center, Houston, TX, United States; ^7^ Department of Molecular & Cellular Oncology, The University of Texas MD Anderson Cancer Center, Houston, TX, United States

**Keywords:** LUAD, K-ras, stat3, DC, Th1, tumor-promoting inflammation

## Abstract

**Introduction:**

K-ras mutant lung adenocarcinoma (KM-LUAD) is a difficult-to-treat cancer subtype in which chronic inflammation pervades the tumor immune microenvironment (TIME). Pro-inflammatory pathways dampen the response to treatments, including immune checkpoint inhibitors, necessitating therapies that target this inflammatory signaling network in the TIME. One of the lynchpins of chronic inflammation in KM-LUAD is signal transducer and activator of transcription 3 (STAT3).

**Methods:**

Here, we tested the anti-tumor and early immunotherapeutic efficacy of TTI-101, a selective small-molecule inhibitor of canonical STAT3 signaling, in a K-ras^G12D^ mutant lung cancer mouse model (CC-LR).

**Results:**

Treatment of CC-LR mice with TTI-101 resulted in reduced tumor burden while increasing dendritic cell (DC) and T helper 1 (Th1) infiltration into the TIME. TTI-101 treatment decreased pY-STAT3 expression in tumors with accompanying increases in several NF-κB anti-tumor target genes including CXCL9, a chemokine for primed T cells. Transcriptional profiling of the TIME revealed improved immune activation and anti-tumor skewing, as well as B cell signaling enrichment. Analysis of human LUAD data demonstrated negative correlations between *STAT3* and Th1/DC infiltration, with DC infiltration also conferring improved survival in LUAD patients with low *STAT3*.

**Discussion:**

Our results highlight the importance of STAT3 in driving early tumorigenesis and offer a preventative treatment window for high-risk individuals and patients with early-stage KM-LUAD.

## 1 Introduction

A quarter of global cancer-related deaths are attributable to lung cancer, of which 40% are histologically classified as lung adenocarcinoma (LUAD) (1–3). A large plurality (25%) of LUAD cases feature a driver mutation in *K-ras*, earning the moniker *K-ras* mutant LUAD (KM-LUAD) (3). Patients with KM-LUAD, especially those with a history of cigarette smoking, carry a poor prognosis, with *K-ras* mutations leading to hyperactive RAF-MEK-ERK signaling and unchecked cellular proliferation (2, 4, 5). While direct inhibition of K-ras has been attempted with some recent success, resistance mechanisms eventually appear, reversing any initial benefits (6, 7). As a result, new therapeutic strategies independent of K-ras signaling are urgently needed to provide better care for an exceedingly large cohort of lung cancer patients.

One promising treatment modality lies in targeting tumor-promoting inflammation. Tumor-promoting inflammation occurs when an initial anti-tumor immune response shifts from acute to chronic, and K-ras is well described as an intrinsic driver of this type of inflammation (8, 9). While the inflammation itself physically damages the lung tissue and promotes tumor proliferation, the flavor of inflammation changes from strongly T helper 1 (Th1) to other non-productive phenotypes such as Th17 and M2-type macrophage polarization (10). We and others have shown the importance of immune cells such as Th17s, myeloid-derived suppressor cells (MDSCs), M2-like macrophages, and neutrophils in lung cancer development via production of cytokines like interleukin 6 (IL-6), IL-17, IL-22, and IL-1β (11–13). In particular, we have shown that targeting IL-6 strongly reduces tumor-promoting inflammation and improves tumor burden in a murine KM-LUAD model (14). The importance of IL-6 signaling has become recognized clinically, with IL-6 neutralizing antibodies currently under study in concert with immune checkpoint inhibitors (NCT04691817).

While targeting IL-6 presents an excellent therapeutic opportunity, this modality is limited to the extracellular space. An alternative strategy to targeting IL-6 is to block key downstream mediators in the pathway, one such being signal transducer and activator of transcription 3 (STAT3). STAT3 plays a key role in pro-tumor machinery across many cell types of the tumor immune microenvironment (TIME). Activated STAT3 within tumor cells has been shown to promote tumor development through transcriptional activation of gene targets that improve cancer survival, stemness, proliferation, and invasiveness (15). In immune cells, STAT3 signaling encourages autocrine production of IL-6 that sustains tumor-promoting inflammation along with immunosuppressive factors such as IL-10 and TGFβ which blunt the anti-tumor response (16). STAT3 activation can occur independently of IL-6 targeting, as it lies downstream to other cytokines including IL-10, IL-22, and IL-27, which are broadly pro-tumor in nature and found in abundance in KM-LUAD (12, 17). Moreover, the use of a small molecule inhibitor enables the targeting of STAT3 in the cytoplasm or nucleus, and such an inhibitor can be upscaled for mass production more easily than a monoclonal antibody (18).

Despite STAT3 representing an attractive therapeutic target, there is disagreement as to the role of STAT3 in early versus late-stage tumors. Most groups have shown that late stage blocking of STAT3 is favorable for tumor clearance (19–28). However, others have shown that targeting STAT3 prior to or during tumorigenesis leads to a loss of epithelial cell identity and a worsening of disease (29, 30). To elucidate the role of STAT3 in early KM-LUAD development, we tested a selective small molecule STAT3 inhibitor, TTI-101, currently in phase II clinical trials, including treatment of idiopathic pulmonary fibrosis and hepatocellular carcinoma (NCT05671835 and NCT05440708, respectively), in a mouse model of KM-LUAD called CC-LR (31). Here, we profile the immune cells, cytokines, chemokines, and transcriptome of the TIME in CC-LR mice to illustrate the effects of STAT3 inhibition across a wide array of cell types. Mice treated during the initial stages of tumorigenesis displayed significant reduction in tumor burden, reprogramming of the TIME chemokine profile, and augmented dendritic cell (DC) and Th1 responses, supporting the hypothesis that STAT3 contributes to early-stage KM-LUAD development.

## 2 Methods

### 2.1 Cell culture

MDA-F471 cells, a *Gprc5a^-/-^* Kras-mutant LUAD cell line with STAT3 hyperactivation, were developed as previously described (32). Cells were cultured in complete growth medium consisting of DMEM (GenDEPOT) supplemented with 10% fetal bovine serum (FBS) (GenDEPOT) and 1% penicillin-streptomycin (GenDEPOT) and maintained at 37°C in a humidified atmosphere with 5% CO_2_. Cells were passaged upon reaching 80-90% confluency. Regular mycoplasma testing was provided by the MD Anderson Cytogenetics and Cell Authentication Core.

Cell viability was measured by MTT assay. Cells were seeded in 96-well plates at a density of 5,000 cells per well in 100 µL of complete growth medium and incubated overnight at 37°C in a 5% CO_2_ atmosphere in serum-free medium containing TTI-101 diluted serially 1:1 from 100-12.5 μM in DMSO; equivalent volumes of DMSO were added to vehicle control wells. After treatment, 10 µL of MTT solution (5 mg/mL in PBS) were added to each well to achieve a final concentration of 0.5 mg/mL. Wells of each concentration were set up in triplicate. Plates were incubated for 4 hours at 37°C to allow for formazan formation. Following incubation, medium was removed and replaced with 100 µL of DMSO to dissolve formazan crystals. The plates were gently shaken for 10–15 minutes to ensure complete dissolution. Absorbance was measured at 570 nm using a microplate reader, with a reference wavelength of 630 nm for background correction. Cell viability was calculated by normalizing absorbance values to untreated control wells.

### 2.2 Mouse husbandry and treatments

CCSP^Cre^/LSL-K-ras^G12D^ (CC-LR) mice were generated as previously described (14). Briefly, C57BL/6 background mice with the LSL-K-ras^G12D^ allele were crossed with mice with Cre recombinase under the control of the club cell secretory protein (CCSP) locus. The resulting mice gain expression of mutant K-ras in the lung epithelium, leading to spontaneous tumor formation that parallels KM-LUAD pathology. All mice were housed under specific pathogen–free conditions and handled in accordance with the guidelines of the IACUC of MD Anderson Cancer Center. Mice were monitored daily for evidence of disease or distress. Both male and female mice were used in this study, and data were analyzed to check for sex-specific effects, which were not seen.

TTI-101 was provided by Dr. David J Tweardy under an approved material transfer agreement with Tvardi Therapeutics, Inc. Mice receiving TTI-101 intraperitoneally (i.p.) were given 100 mg/kg TTI-101 in DMSO five times weekly from 10-to-14 weeks of age (N = 8 controls, 9 TTI-101-treated). Oral gavage (o.g.) delivery of TTI-101 was formulated at 50 mg/kg in Labrasol: PEG 400 at a ratio of 3:2 and given once daily from 10-to-14 weeks of age (N = 21 controls, 25 TTI-101-treated). Mice were weighed daily to determine drug dose and to monitor for weight loss, a possible sign of toxicity or poor gavage technique. Dosing was performed as previously described (33, 34).

### 2.3 Mouse necropsy, bronchoalveolar lavage, plasma, and histology preparation

To harvest samples, 14-week-old CC-LR mice were anesthetized by i.p. injection of 0.8 mL of 0.25 mg/mL 2,2,2-tribromoethanol (Avertin, Sigma). Cannulas (Luer) were inserted into the tracheas and sutured into place, and lung surface tumors were then counted.

For bronchoalveolar lavage fluid (BALF) collection, a portion of mice received two sequential instillations of 1 mL of PBS via the cannulas. Blood was collected from the inferior vena cava and centrifuged at 2,370 x G for 5 minutes, and supernatants were collected as plasma. The lungs of these mice later underwent perfusion with 10 mL of PBS injected into the right ventricle. The lung tissue was then snap frozen in liquid nitrogen for downstream RNA and protein analyses. BALF total white blood cell (WBC) count was then obtained on a hemocytometer. Cytocentrifugation and subsequent Wright-Giemsa (Sigma) staining were performed to measure major immune cell lineages.

For histology preparation, a portion of mice had blood drawn and were perfused as described above, after which the lungs were inflated with 10% buffered formalin (Sigma) for 10 minutes via the cannulas. Lungs were removed and stored in formalin for 24 hours, followed by 24 hours in PBS. Lungs were then passed through an automated tissue embedder (Leica Biosystems) to make formalin fixed paraffin embedded (FFPE) blocks.

### 2.4 Quantification of TTI-101 drug concentrations in plasma

Plasma collected as above was mixed with stabilizer solution containing 20 mg/mL of NaF, 25 mg/mL of Na2SO3, and 25 mg/mL of L-ascorbic acid in ddH2O with 1X PBS at a 1:1 ratio. Aliquots of plasma from mice were spiked with 5ul of deuterated TTI-101(D7) as the internal standard (IS) to a final concentration of 5 μg/mL. Calibration standards and QC samples of TTI-101 were prepared by spiking 5 μL of the relevant working solutions of TTI-101 and SI into 100 μL of blank normal plasma. TTI-101 was extracted through one-step liquid-liquid extraction (LLE) using methyl tert-butyl ether (MTBE). Samples were reconstituted in 100 μL of methanol before analysis. The LC-MS/MS analysis was performed on a QTRAP 5500 Sciex hybrid quadrupole-linear ion trap system with a turbo ion spray source coupled to a Sciex LC Exion liquid chromatography system. Data acquisition and quantification were conducted using Analyst 1.6. (Redwood City, CA, USA). Chromatographic separation was achieved using a Synergi™ 4 µm Fusion-RP 80 Å, LC Column 50 x 2 mm at a temperature of 40°C with a 3 min linear gradient at 500 μL/mL. The aqueous mobile phase (solvent A) was created as follows: 0.1% (vol/vol) formic acid and 5 mM ammonium acetate in ddH2O. The organic phase (solvent B) was 0.1% formic acid and 5 mM ammonium acetate in methanol. MRM monitoring in positive mode (ESI+) was used to detect TTI-101 and the IS, with m/z 472.093 > 301.1 for TTI-101 and m/z 479.200 > 301.1 for IS. The calibration curve for TTI-101 was generated from the peak area ratio of TTI-101 to the peak area of its internal standard TTI-101(d7) using the Linear regression analysis with 1/X weight over the range of 0.03-30uM. All LC-MS/MS reagents including methanol, water, ammonium acetate, and formic acid were obtained from Honeywell Fluka (Morris Plains, NJ). Methyl tert-butyl ether (MTBE) was obtained from Sigma Aldrich. The C18 Synergi™ 4 µm Fusion-RP 80 Å LC column (50 × 2 mm) was purchased from Phenomenex, (Torrance, CA, USA).

### 2.5 Tissue histology

Hematoxylin and Eosin (H&E) staining was performed on multiple 5 μm-thick cuts from FFPE lungs, with individual sections separated by at least 50 μm. Slides were scanned at 4X magnification and stitched together on a BZ-X810 microscope (Keyence). Normal lung and lesion areas were manually annotated in ImageScope 12.4.3 (Leica Biosystems).

Immunohistochemistry (IHC) for Ki-67 (1:200, ab16667, Abcam) and pY-STAT3 (Tyr705) (1:200, 9145S, Cell Signaling Technology) were performed as previously described (14). Ki-67^+^ and pY-STAT3^+^ nuclei within tumors were quantified using ImageJ (NIH) as previously described (35). Additionally, pY-STAT3 staining and lesion type in o.g. CC-LR mice was quantified using HALO Indica Labs image analysis platform (version 3.5.3577.108).

Multiplex immunofluorescence staining (Lunaphore COMET) was performed on FFPE-derived sections as described in Wang et al. (36). Antibodies against major immune markers were sequentially stained, imaged, and inactivated to build multicolor image overlays. Images were imported into Visiopharm for segmentation, gating, and quantification (see **Supplementary Table 1** for full antibody panel and cell segmentation logic).

### 2.6 RNA extraction and qRT-PCR analysis

Mouse lung RNA was extracted by placing cryopreserved lung tissue into tubes containing chrome plated steel beads and QIAzol (Qiagen). Tubes were then shaken on a Mini-BeadBeater 16 (BioSpec) for 10–20 seconds twice, incubating samples on ice between each round. Homogenates were then passed through a QIAshredder (Qiagen) for further cellular lysis. RNA extraction was then performed using the RNeasy Mini kit (Qiagen) following the manufacturer’s instructions. cDNA was made using the qScript cDNA SuperMix (Quanta Biosciences). qRT-PCR was run using SYBR Green FastMix (Quanta Bioscience) on a CFX96 Touch™ Real-Time PCR Detection System (Bio-Rad). Beta-actin (*Actb*) or CD45 (*Ptprc*) were used as housekeeping genes, with results presented as fold change using the ΔΔCt method. Primers used are listed in **Supplementary Table 2**.

### 2.7 Protein extraction and analysis

Mouse lung protein was extracted by homogenizing cryopreserved lung tissue in tubes containing chrome plated steel beads. For total protein, RIPA buffer (Sigma) with protease + phosphatase inhibitor cocktail (Thermo Fisher Scientific) was used; for nuclear and cytosolic fractionation, buffers from the NE-PER Nuclear Protein Extraction Kit (Pierce) were used. After incubating for 30 minutes on ice, samples were centrifuged at 18,620 x G for 30 minutes at 4°C, with supernatants collected. Protein concentration was determined using a bicinchoninic acid assay (Thermo Fisher Scientific) according to the manufacturer’s instructions.

BALF supernatants were analyzed by Olink Proximity Extension Assay using the Olink Target 48 (Olink Proteomics) panel for mouse immune markers. Samples were run by the Houston Methodist Research Institute Immunoediting Core according to the manufacturer’s instructions. Data were provided as NPX values (normalized protein expression with log2-type transformation) and pg/mL (absolute quantification).

Immunoblotting was performed as previously described (37), with nuclear fractions used for STAT3 detection and total protein used for p65. Primary antibodies were against STAT3 (1:1000; catalog 4904S, Cell Signaling Technology), p-p65 (Ser536) (1:1000, catalog 3033S, Cell Signaling), total p65 (1:1000, catalog ab32536, Abcam), histone H3 (1:1000; catalog 4499S, Cell Signaling Technology), and β-actin (1:1000; catalog 4970S, Cell Signaling Technology).

The NF-κB (p65) binding activity was measured using the NF-κB (p65) Transcription Factor Assay Kit (Cayman Chemical Company) as previously described (13), with 10 μg of nuclear extracts run in duplicate. Optical density (OD) was measured at 450 nm, and fold binding activity was calculated by normalizing to control ODs.

### 2.8 Flow cytometry

Flow cytometry was performed as previously described (13): Briefly, lungs were harvested as mentioned above but were then manually cut using scissors into a paste and then digested for 30–45 minutes at 37°C in 1 mg/mL collagenase IV (Gibco) in RPMI (GenDEPOT). Digested samples were then mechanically dissociated into single-cell suspensions using 70 μm nylon mesh (Falcon) and subjected to RBC lysis. Cells were then resuspended in FACS buffer (1X PBS, Sigma; 2 mM EDTA, Millipore Sigma; 1% FBS, GenDEPOT) for staining. 3 x 10^6^ cells per sample were allocated to separate myeloid and lymphoid panels (see **Supplementary Table 3** for complete antibody list and panel composition). Myeloid samples underwent Fc blocking with anti-CD16/CD32 (clone 2.4G2, Tonbo) concurrent with surface staining for 30 minutes on ice, followed by fixation with 1% formaldehyde. Lymphoid samples underwent intracellular cytokine stimulation: cells were cultured in RPMI (GenDEPOT) containing 50 ng/mL phorbol 12-myristate 13-acetate (PMA, Sigma), 500 ng/mL ionomycin (Sigma), 1 μL/mL GolgiStop (BD Biosciences), and 0.7 μL/mL GolgiPlug (BD Biosciences) for 4–6 hours at 37°C. After surface staining, cells were permeabilized with the FoxP3 Transcription Factor Staining Kit (Invitrogen) for intracellular/intranuclear markers. All data were acquired using an LSRFortessa X-20 (BD) and analyzed with FlowJo software, version 10 (Tree Star). Gating strategies are shown in **Supplementary Figure 5**.

### 2.9 Bulk RNA sequencing and GSEA

Following RNA extraction as described above, whole-transcriptome sequencing (RNA-seq), library construction, and gene set enrichment analysis (GSEA) were performed by Novogene (Beijing, China) using standard protocols, including quality control, library preparation with poly(A) selection, and paired-end sequencing on the Illumina platform. Data were processed and analyzed following Novogene’s recommended RNA-seq pipeline. GSEA results were generated by comparison with GO Enrichment gene sets.

### 2.10 Cellular deconvolution and TCGA analysis

RNA-seq immune profiles were deconvoluted from bulk RNA-seq data using the xCell (38) and CIBERSORT (39) algorithms in R (version 3.5.1; R Project for Statistical Computing). Gene expression data from LUAD patients, including both males and females, were obtained from TCGA (https://portal.gdc.cancer.gov). Linear regression was run to correlate immune cell infiltration with *STAT3* expression. Overall survival was calculated for patients compared to B cell infiltration, and overall survival for patients with low *STAT3* expression was compared to DC infiltration.

### 2.11 Statistics

Data are presented as mean ± SEM. Comparisons between control and TTI-101-treated groups were calculated by a 2-tailed t test, with P < 0.05 being statistically significant. All statistical analyses were performed in GraphPad Prism (Version 10.3.1).

### 2.12 Data availability statement

Data were generated by the authors and are available on request.

## 3 Results

### 3.1 TTI-101 reduces growth of tumor cells in KM-LUAD models

To test the effectiveness of TTI-101 on *K-ras* mutant cancer cells, we first treated a mouse-derived KM-LUAD cell line with increasing concentrations of TTI-101. The MDA-F471 mouse LUAD cell line, which is *K-ras* mutant and STAT3-addicted (32), demonstrated a dose-dependent decrease in viability upon treatment with TTI-101, with an IC_50_ of 14.74 μM (**Supplementary Figure 1A**).

We next examined TTI-101 *in vivo* using a KM-LUAD mouse model possessing *K-ras^G12D^* expression under the control of club cell secretory protein (CC-LR). TTI-101 was formulated in DMSO and delivered intraperitoneally (i.p.) at 100 mg/kg five days per week from 10-to-14 weeks-of-age. This treatment timeline was chosen in order to test for any tumor-preventative effects, since adenoma formation is evident at 14 weeks of age in the CC-LR model (40). Fourteen-week-old TTI-101-treated mice displayed significant reduction in tumor burden as measured by surface tumor number (**Supplementary Figure 1B**) and the ratio of tumor to healthy lung area by histology (**Supplementary Figures 1C, D**). Tumors from these mice showed reduced levels of activated STAT3 (STAT3 phosphorylated on Y705; pY-STAT3) using both pY-STAT3 immunohistochemistry (IHC; **Supplementary Figures 1E, F**) and Luminex bead-based assays (**Supplementary Figure 1G**).

**Figure 1:**
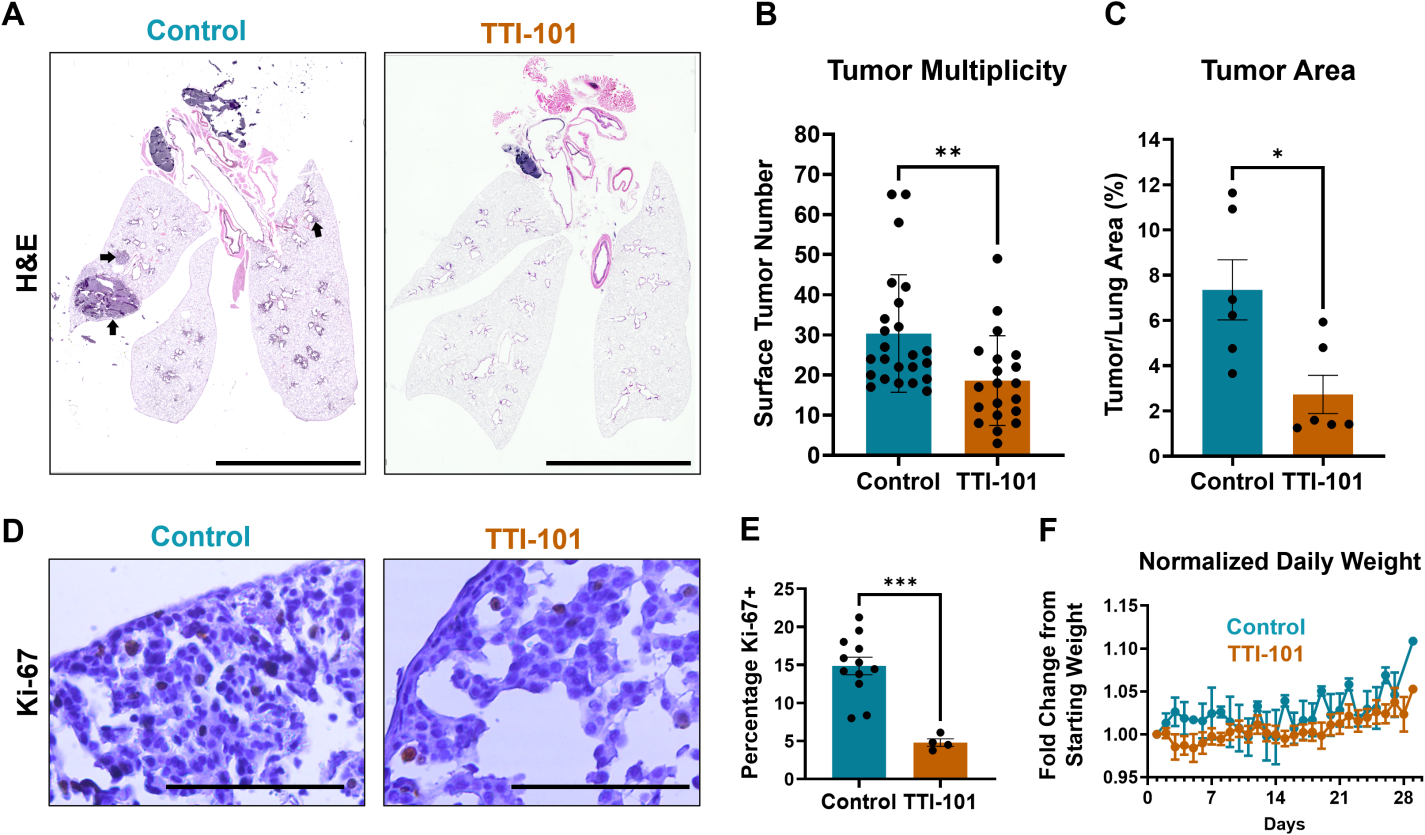
STAT3 inhibition reduces development and growth of lung tumors. **(A)** Representative stitched photomicrographs of H&E-stained sections of 14-week-old CC-LR mouse lungs either untreated or given TTI-101 by oral gavage (o.g.); arrows indicate representative tumor areas; original magnification, 4X; scale bar is 5 mm. **(B)** Tumor burden as measured by manual counting of lung surface tumors (N = 21-25). **(C)** Percentage of tumor area over total lung area (N = 6). **(D)** Representative photomicrographs of Ki-67-stained sections and quantified in **(E)** (N = 4-12); scale bar is 200 μm. **(F)** Weight, normalized to initial weight, of CC-LR mice given 50 mg/kg TTI-101 o.g. from 10-to-14 weeks-of-age. Data represent mean ± SEM. ***P < 0.001, **P < 0.01, *P < 0.5 by unpaired *t* test.

To facilitate its potential use in patients, we next examined the effect of TTI-101 treatment (50 mg/kg once daily) in CC-LR mice again from 10-to-14 weeks of age using TTI-101 formulated in Labrasol/PEG-400 that allowed for its administration by oral gavage (o.g.) (41). Plasma TTI-101 levels reached 1952 +/- 914.5 ng/mL in treated mice and were below the lower limit of quantitation (LLOQ) for controls (**Supplementary Table 4**). Importantly, we witnessed a 39% reduction in tumor burden (p = 0.0053; **Figures 1A–C**). In addition, the number of Ki-67^+^ tumor cell nuclei was decreased, indicating reduced tumor cell proliferation (**Figures 1D, E**). As previously demonstrated, TTI-101 was well tolerated with no evidence of toxicity or weight loss observed (**Figure 1F**).

### 3.2 STAT3 inhibition reduces STAT3 signaling while skewing to an NF-κB-driven chemokine profile

**Figure 2:**
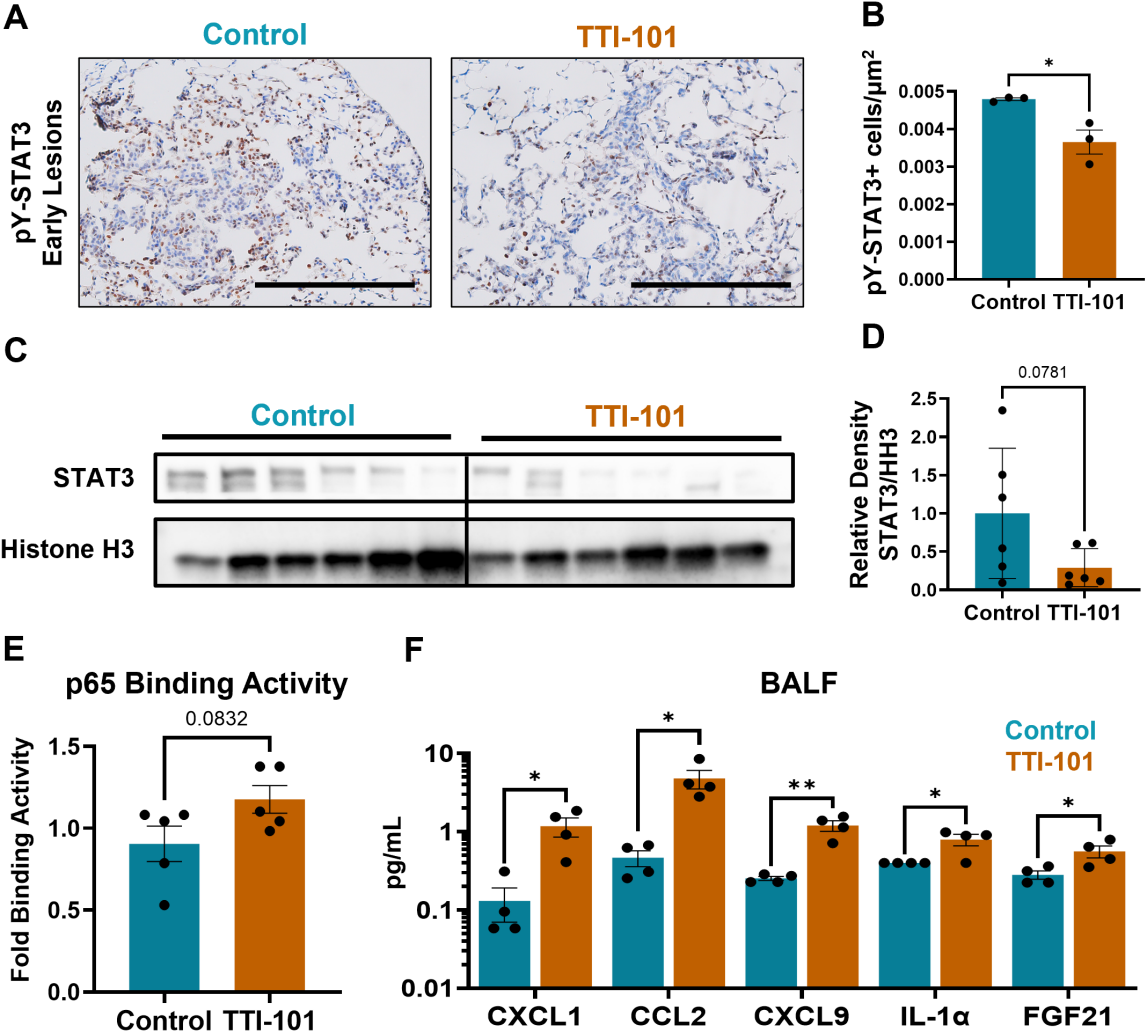
STAT3 inhibition reduces STAT3 signaling intensity while skewing to an NF-κB-driven chemokine profile. **(A)** Representative photomicrographs of pY-STAT3-stained sections in early lesions in CC-LR mice (N = 3); magnification, 20X; scale bar is 200 μm. **(B)** Quantification of pY-STAT3^+^ cells from CC-LR mice by μm^2^ of lesion area. **(C)** Immunoblot of STAT3 and histone H3 (HH3; loading control) in nuclear fractionated lung tissue lysates, quantified in **(D)** as relative density of STAT3 to HH3 (N = 6). **(E)** p65 DNA binding assay, with fold binding activity calculated based on OD of control samples (N = 5). **(F)** Olink protein extension assay analytes from bronchoalveolar lavage fluid (BALF) (N = 4). Data represent mean ± SEM. **P < 0.01, *P < 0.5 by unpaired t test.

To determine if STAT3 signaling was impacted by TTI-101, we measured STAT3 in CC-LR mice treated with TTI-101 by o.g. Early lesions displayed a lower density of pY-STAT3^+^ cells (**Figures 2A, B**), and immunoblotting of nuclear fractions from whole lung protein showed decreased translocation of STAT3 into the nucleus (**Figures 2C, D**). Since there is known modulation between STAT3 and NF-κB signaling (42), we performed immunoblotting to assess for NF-κB pathway components. However, we saw no changes in protein abundance of the NF-κB nuclear effector subunit p65 (**Supplementary Figures 2A, B**). To check for possible changes in transcriptional activity, we performed a p65 DNA binding assay, which measures the ability of the NF-κB p65 subunit to bind its transcriptional target sequences. Using this method, we saw a trend for increased p65 DNA binding (**Figure 2E**).

To assess changes in the TIME and any downstream effects of STAT3 inhibition, we performed an Olink Proximity Extension Assay on bronchoalveolar lavage fluid (BALF) from CC-LR mice. BALF was chosen as a means of biomarker discovery and facilitating clinical translation because cytokine changes in BALF are more reflective of the secretory products of airway immune cells. Olink of BALF revealed upregulation of various soluble mediators (**Figure 2F**): Three chemokines, CXCL1, CCL2, and CXCL9, were upregulated, which provide chemotactic impetus to neutrophils, monocytes, and primed T cells respectively (43–45). IL-1α, a pro-inflammatory cytokine (46), was also upregulated. These four markers are notable as being transcriptional targets of NF-κB (47), although CXCL9 is also strongly induced by IFNγ (48). Normalized protein expression (NPX) data from Olink showed upregulation of other NF-κB targets: CCL22, CSF1, CSF2, CSF3, CXCL2, and TNFα (**Supplementary Figure 2C**). We also noted an increase in FGF21, a factor which promotes cholesterol metabolism and is known to cause exhaustion in T cells (49).

Collectively, these results indicate a possible shift in lung tumors from STAT3- to NF-κB-driven inflammation, with the reprogramming of the TIME evidenced primarily by changes in chemokine composition rather than prolific cytokine profile alterations.

### 3.3 STAT3 inhibition increases DC and Th1 proportion within the TIME

**Figure 3:**
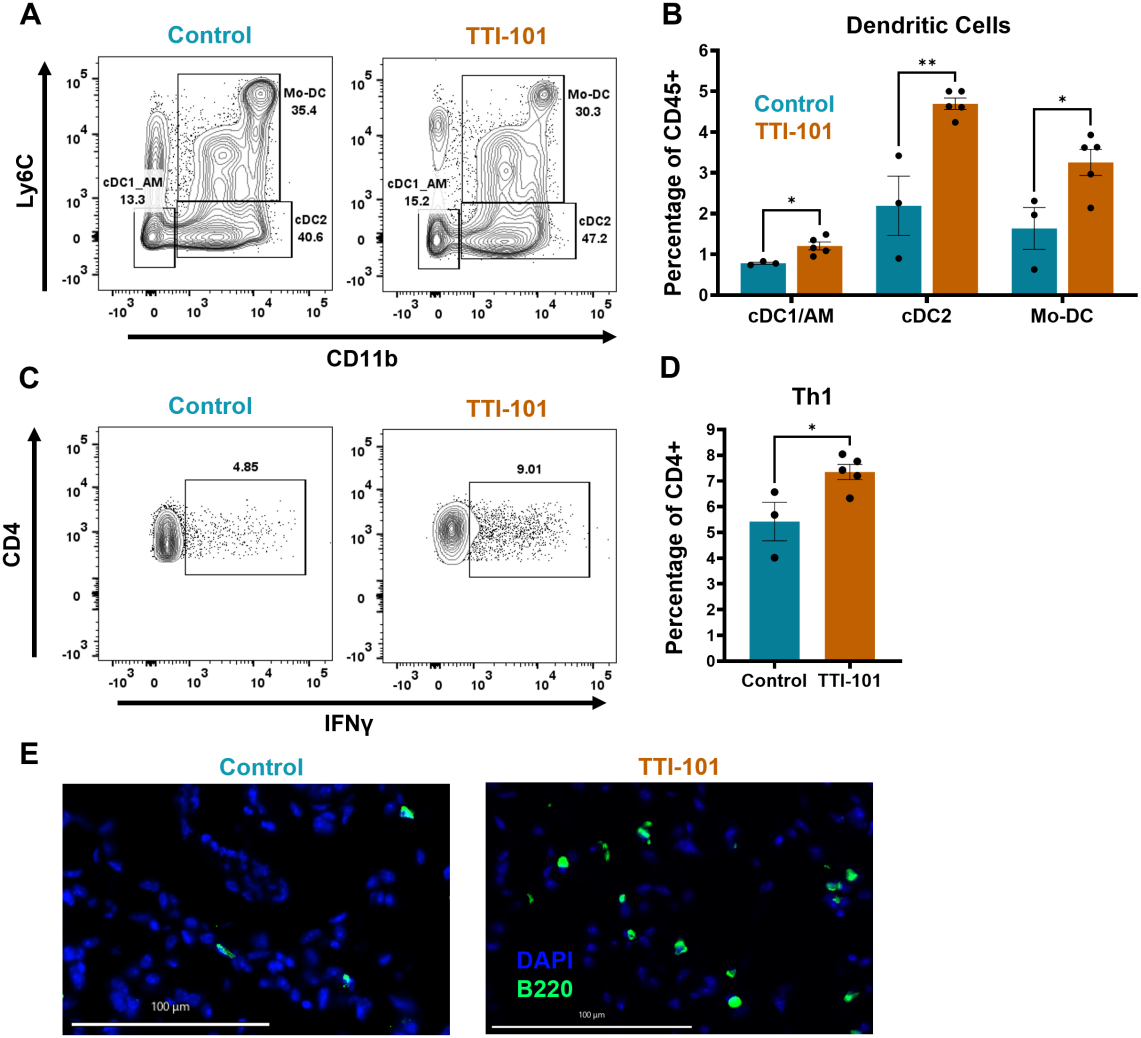
STAT3 inhibition increases DC and Th1 proportion within the TIME. **(A)** Representative dot plots of myeloid cell flow cytometry analysis pre-gated on CD11c^+^ cells; expression of Ly6C and CD11b is used to delineate classical type 1 DCs (cDC1s)/alveolar macrophages (AMs), cDC2s, and monocytic DCs (Mo-DCs), and is quantified in **(B)** as a percentage of CD45^+^ cells (N = 3-5). **(C)** Representative dot plots of lymphoid cell flow cytometry analysis with *ex vivo* restimulation by PMA/Ionomycin, pre-gated on CD3^+^ cells; expression of CD4 and IFNγ is used to mark T helper 1 (Th1) cells and is quantified in **(D)** as a percentage of CD4^+^ cells (N = 3-5). **(E)** Representative multiplex immunofluorescence (COMET) images for B cells (B220^+^); original magnification, 20X; scale bar is 100 µm. Data represent mean ± SEM. **P < 0.01, *P < 0.5 by unpaired *t* test.

To better understand changes in the immune cell populations within the TIME, we performed flow cytometry of CC-LR mouse lungs treated with TTI-101 by o.g. In the myeloid compartment, the most significant changes were seen in dendritic cells (DCs), with classical type 1 (cDC1), classical type 2 (cDC2), and monocytic DC (Mo-DC) proportions elevated in the lung (**Figures 3A, B**). It should be noted, however, that our myeloid gating strategy was unable to satisfactorily delineate alveolar macrophages (AMs) and cDC1s, as both are CD11b^-^ CD11c^+^. Therefore, we treated these cells as a lumped group labeled cDC1/AM. cDC2s displayed trends for increased MHC-II expression (**Supplementary Figure 3C**), indicating activation and antigen presentation ability. Flow cytometry of BALF indicated a trending increase in cDC2 infiltration and Ly6G low immature granulocytes (**Supplementary Figure 3B**), which may have migrated in response to elevated CCL2 and CXCL1 respectively (**Figure 2F**). Immature granulocytes may develop into polymorphonuclear MDSCs (PMN-MDSCs), and these findings will be explored in greater depth later.

Within the lymphoid compartment, we noted an increase in IFNγ-producing CD4^+^ helper T cells (Th1s) (**Figures 3C, D**). IFNγ transcripts (*Ifng*) were significantly elevated as measured by qRT-PCR, and expression of T-bet (*Tbx21*), the defining Th1 lineage transcription factor, trended higher (**Supplementary Figure 3D**). However, no significant changes were seen in other T cell subtypes (data not shown). We also noted a trend for increased B cell proportion by flow cytometry (**Supplementary Figure 3E**), an observation corroborated by immunofluorescence imaging of lung sections showing some heterogeneous increase in B cell infiltration into the lung (**Figure 3E**, **Supplementary Figure 3F**). Taken together, our findings suggest that STAT3 inhibition by TTI-101 augments DC infiltration into the TIME and bolsters the Th1 response.

### 3.4 Transcriptomic profiling indicates STAT3 inhibition broadly increased immune activation, particularly DCs and B cells

**Figure 4:**
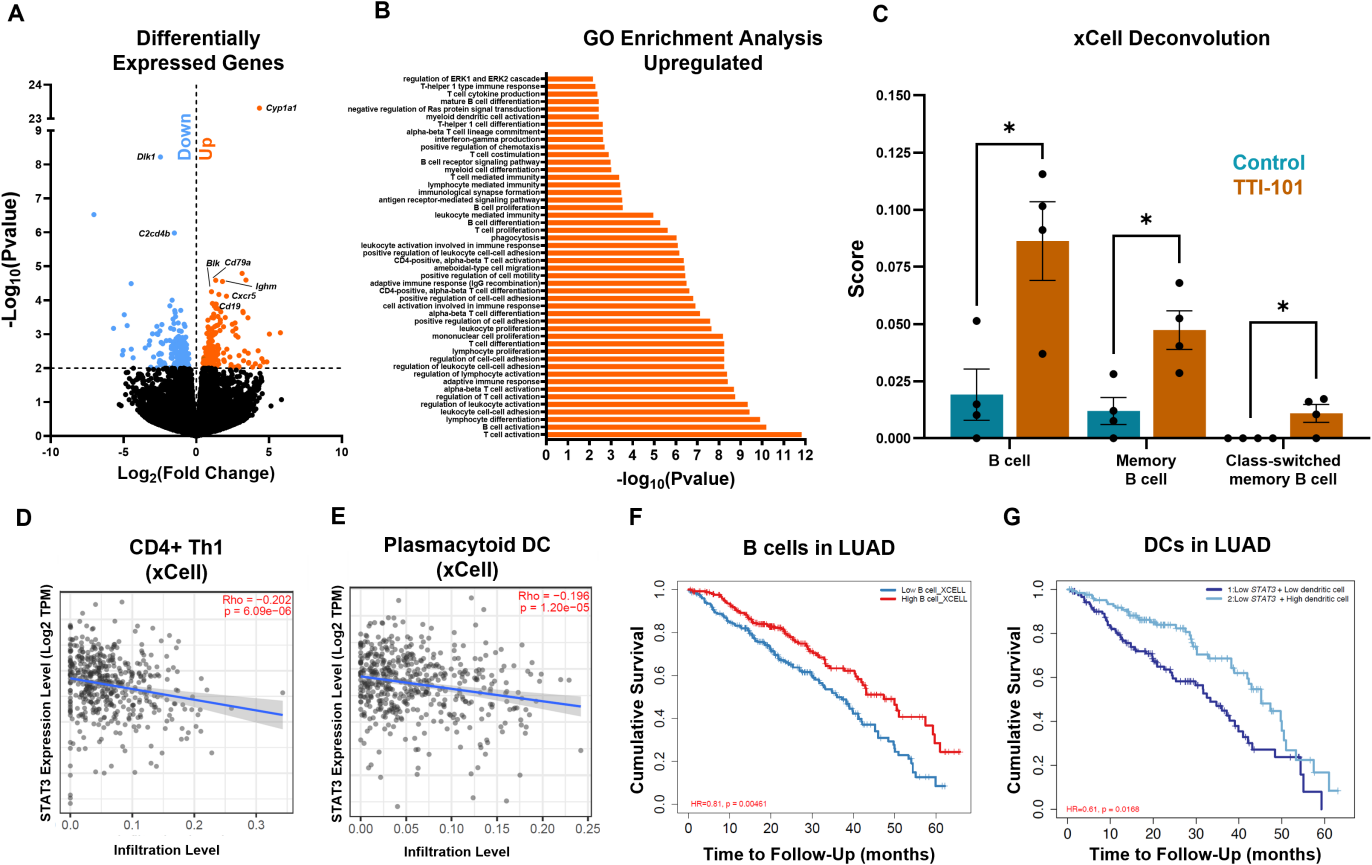
Transcriptomic profiling indicates STAT3 inhibition broadly increases immune activation. **(A)** Volcano plot of upregulated (orange) and downregulated (blue) genes following TTI-101 treatment in CC-LR mice (N = 4). **(B)** Go enrichment GSEA showing the top enriched pathways (N = 4). **(C)** Cellular deconvolution score of RNA-seq data by xCell. Correlation of *STAT3* expression in LUAD patients (adjusted by stage) vs. Th1 **(D)** and plasmacytoid DC **(E)** infiltration (N = 515). Survival curves of B cells in LUAD **(F)** and DCs in LUAD with low *STAT3* expression **(G)**. Data represent mean ± SEM. *P < 0.5 by unpaired *t* test.

To better survey the effects of TTI-101 in our model in an unbiased manner, we performed whole-transcriptome sequencing (RNA-seq) of mRNA from whole lung lysates. Applying a cutoff rate of FDR > 2, we discovered 398 genes that were differentially expressed following STAT3 inhibition (**Figure 4A**). Gene set enrichment analysis (GSEA) by GO enrichment indicated downregulated pathways related to microtubules and cilia (**Supplementary Figure 4A**). Additionally, *C2cd4b*, a marker of acute inflammation (50), and *Dlk1*, a driver of Wnt signaling, lung repair and stemness, and cancer stemness (51–53), were downregulated (**Figure 4A**). However, a larger number of pathways related to immune activation were found to be significantly enriched (**Figure 4B**). Broad T, B, and myeloid cell activation pathways were seen, including cell adhesion, cell migration, B cell receptor (BCR) signaling, immune proliferation, and more. Several transcripts in these pathways were upregulated, including *Blk* (T cell activation) and *Cd79a* (mature B cells) (**Figure 4A**). GSEA also supported our findings of increased Th1/DC activation as well as enrichment of inhibitory gene sets governing Ras and ERK1/2 (**Figure 4B**), indicating that TTI-101 mitigates hyperactive K-ras signaling.

We next deconvoluted the bulk RNA-seq data to quantify immune cell infiltration. Using the xCell deconvolution method (38), we saw significant enrichment in B cells, memory B cells, and class-switched memory B cells (**Figure 4C**). B cell genes were seen to be upregulated, including *Ighm* (Ig mu heavy chain), *Cd19* (B cell lineage), and *Cxcr5* (B cell chemotaxis) (**Figure 4A**). Using xCell, we also noticed a higher score for common lymphoid progenitors (CLPs) (**Supplementary Figure 4B**). Use of CIBERSORT deconvolution (39) revealed a decrease in M2 score (**Supplementary Figure 4C**). Deconvolution scores were correlated with LUAD data from The Cancer Genome Atlas (TCGA): Th1s and plasmacytoid DCs negatively correlated with *STAT3* expression in LUAD tumors (**Figures 4D, E**), a pattern we also noticed for CLPs (**Supplementary Figure 4D**). Owing to the B cell phenotype found in our RNA-seq data, we queried TCGA for LUAD patients and B cell infiltration. Our results showed that higher B cell infiltration led to increased cumulative survival (**Figure 4F**). Since we were operating in the setting of STAT3 inhibition, we narrowed our analysis to patients with low *STAT3* expression. We found that patients with low *STAT3* showed improved survival with higher infiltration of DCs (**Figure 4G**), supporting our finding of increased DC infiltration in CC-LR mice. These data suggest that DC tumor infiltration may serve as a predictor of response to STAT3 inhibition while suggesting a role for B cells in the anti-tumor response engendered by TTI-101.

## 4 Discussion

In this study, we demonstrated that inhibiting STAT3 with TTI-101 is an efficacious means of targeting a myriad of pro-tumor STAT3 functions across the tumor and immune compartments. In CC-LR mice, STAT3 inhibition attenuated tumor burden and tumor proliferation. Immunologically, we observed substantial reprogramming of the TIME: in addition to decreased STAT3 signaling, we observed upregulation of many NF-κB targets, including monocyte and T cell chemokines (CCL2 and CXCL9). Flow cytometry profiling of the lung revealed changes in both the myeloid and lymphoid compartments: DC proportions were higher following treatment with TTI-101, with a corresponding increase in Th1s. Increased immune activation was reinforced by differential gene analysis, with profiles for T, B, and myeloid cell activation showing significant positive enrichment, particularly for B cells. Comparisons with LUAD data from TCGA data likewise indicated inverse relationships between *STAT3* expression and Th1 and DC infiltration. B cell and DC infiltration even correlated with improved survival.

Our results shed light on the temporal nature of STAT3 in lung tumorigenesis. Some studies have reported that early *Stat3* ablation is pro-tumorigenic (29, 30). These studies showed that deletion of *Stat3* in the lung epithelium concurrent with K-ras^G12D^ induction fostered KM-LUAD development, growth, and lethality. The temporal nature of STAT3 function was illustrated by Zhou et al., who demonstrated that preemptive targeting of STAT3 prior to carcinogen exposure resulted in increased K-ras mutation rate, inflammation, and tumorigenesis. However, once tumors were established, *Stat3* deletion proved integral to reducing tumor growth (26). In our hands, we were able to pharmacologically target STAT3 after initial tumor formation but saw decreased tumor growth. Therefore, we conclude that STAT3 inhibition is a viable means of treating KM-LUAD between initial tumor formation and tumor establishment.

Additionally, our results show that targeting STAT3 does not only affect tumor cells but repolarizes the TIME to an anti-tumor phenotype. Skewing from STAT3 to NF-κB signaling may explain the resulting changes in secreted factors. The NF-κB target CCL2, which is a known monocyte chemokine, is also a DC chemotactic factor (54) and may well explain the increased abundance of DCs within the lung. Likewise, CXCL9 is known to recruit primed T cells to sites of inflammation (45) and may correspond with our finding of increased Th1 prevalence.

DCs are well known to play an essential role in priming T cell-mediated anti-tumor immune responses (55, 56), and the trend for cDC2s to upregulate their MHC-II expression is indicative of increased activation and antigen presentation (57). While STAT3 is known to play an important role in DC maturation (58, 59), DC-specific knockouts of STAT3 in mice result in DCs that are fewer in number but greater in anti-tumor function and Th1 priming ability via IL-12 secretion (20, 60). Therefore, we suspect that TTI-101 enhances DC antigen presentation and T cell priming to elicit an anti-tumor response.

Th1 function is likewise influenced by STAT3 signaling: STAT3-derived IL-6 and TGFβ drive CD4 differentiation away from Th1 towards Th17, a CD4 subtype that is often pro-tumorigenic through IL-17-mediated recruitment of MDSCs (61–64). STAT3 likewise is important for regulatory T cell (Treg) differentiation via TGFβ (65), and deletion of STAT3 in the murine T cell compartment obliterates Treg tumor infiltration (66). In our hands, STAT3 inhibition using TTI-101 in the CC-LR model seems to promote Th1 function.

Evidence of anti-tumor synergy between myeloid and T cells is also mirrored in our RNA-seq results, with numerous anti-tumor immune signaling pathways showing significant enrichment. It is also telling that in LUAD tumors with low *STAT3* expression, DC infiltration is predictive of survival. In subsequent studies, we will focus more on the DC:T interaction and the priming process in response to TTI-101.

It is worth noting that while we only saw a trending increase in B cell abundance in the lung, transcriptomic profiling indicated increased BCR signaling and B cell activity, and cellular deconvolution predicted increased B cell enrichment. Humoral immunity and tertiary lymphoid structures (TLSs) are known positive prognostic indicators in KM-LUAD (67, 68); while we saw no evidence of TLSs (data not shown), it is likely that humoral immunity plays a role in the response to TTI-101 and merits further investigation.

We also detected TTI-101-induced gene transcript changes that suggest potential pathways for the emergence of TTI-101 resistance. For example, FGF21, which inhibits CD8 T cells by boosting cholesterol metabolism (49), was upregulated in BALF and may herald blunting of the anti-tumor responses. Similarly, increased CXCL1, a known neutrophil chemokine (43), could lead to recruitment of PMN-MDSCs, which are immunosuppressive and a negative prognostic indicator in KM-LUAD (10).

While this study demonstrated the efficacy of STAT3 inhibition via TTI-101 in reducing tumor burden and in reprogramming inflammation in KM-LUAD, several limitations should be addressed. First, we have studied the role of TTI-101 in a preventative setting in CC-LR mice. Our early treatment regimen is most translatable to high-risk patients who, through improved early screening, may benefit from a prophylactic intervention. However, providing the drug once tumors are established or increasing drug exposure may yield different results, and we plan to study STAT3 inhibition at later timepoints moving forward. Second, although we observed increased immune activation, including DC and Th1 responses, the precise mechanism of immune priming and the durability of this response remain unclear. While we see tumor-intrinsic effects of STAT3 inhibition in terms of reduced tumor cell proliferation, we do not see changes in angiogenesis or apoptosis markers (data not show). This suggests that tumor-extrinsic STAT3 function in DCs and T cells play a marked role. In the same vein, we cannot precisely define if one DC subset or another is playing a greater role, and we cannot rule out a role for AMs, since they were indistinguishable from cDC1s in our gating. Additionally, while transcriptomic analyses suggested increased B cell enrichment and activity, our flow and COMET results are limited and only trending. The functional contribution of humoral immunity and its role in the anti-tumor response in this model warrant further investigation. Moreover, the CCR2/CCL2 pathway recruits mononuclear MDSCs (M-MDSCs) into tumors, in addition to monocytes and DCs (69).

We will focus future studies on longitudinal monitoring of M- and PMN-MDSC recruitment to further dissect the interplay between MDSCs, DCs, T cells, and B cells in shaping anti-tumor immunity. Since the use of TTI-101 alone did not completely abrogate lung tumor burden, and since TTI-101 did not appear to alter CD8 number or their cytokine profiles (data not shown), we plan to combine TTI-101 with anti-PD-1 to see if there are synergistic effects that could boost cytotoxicity. In addition, it will be interesting to test combination therapy with K-ras inhibitors, conventional chemotherapy, and other immunotherapeutic interventions such as DC vaccination to improve DC function and T cell priming. As this study was limited to the early-stage lung tumor setting, we also plan to study the effects of TTI-101 in later-stage tumors alone and in combination with the other therapies mentioned.

In conclusion, our findings establish STAT3 inhibition via TTI-101 as a promising early therapeutic/preventative approach for KM-LUAD that can reduce tumor burden, reprogram the TIME, and enhance anti-tumor immunity.

## Data Availability

The original contributions presented in this article are deposited in the GEO repository, accession number GSE294807. The individual samples are stored in GSM8920639-GSM8920646.
