# Supplementary material for: Selective inhibition of canonical STAT3 signaling suppresses K-ras mutant lung tumorigenesis and reinvigorates anti-tumor immunity
Source: Front Immunol. 2025 Apr 28;16:1575181. doi: 10.3389/fimmu.2025.1575181 (PMC12066534; doi:10.3389/fimmu.2025.1575181)
Supplement: Supplementary file 1 [file DataSheet1.pdf]

Supplementary Material

1 Supplementary Figures

1.1 Supplementary Figure 1

Matched with Figure 1. **(A)** MTT viability assay of MDA-F471 cells, comparing various concentrations of TTI-101 vs. an equivalent volume of DMSO vehicle (control); performed in triplicate. **(B)** Tumor burden of 14-week-old CC-LR mice given 100 mg/kg TTI-101 intraperitoneally (i.p.) from 10 to 14 weeks-of-age as measured by manual counting of lung surface tumors (N=8-9). **(C)** Representative photomicrographs of H&E-stained sections, quantified in **(D)** (N = 3); magnification, 4X; scale bar is 0.5 mm. **(E)** Representative photomicrographs of IHC for p-STAT3-stained sections, quantified in **(F)** (N = 20-30); magnification, 20X; scale bar is 200 µm. **(G)** Luminex bead assay for pY-STAT3 and total STAT3, expressed as the ratio of pY-STAT3/total STAT3. Data represent mean ± SEM. ****P* < 0.001, ***P* < 0.01, **P* < 0.5 by unpaired *t* test.\

1.2 Supplementary Figure 2

Matched with Figure 2. **(A)** Immunoblot of phospho-p65 (p-p65), total p65, and Ponceau S (total protein loading control), quantified in **(B)** as density of p-p65 to Ponceau S (N = 6-7). **(C)** All analytes run by Olink PEA shown in units of normalized protein expression (NPX) (N = 4); an NPX reading of zero or negative value is denoted by 0. Data represent mean ± SEM. ****P* < 0.001, ***P* < 0.01, **P* < 0.5 by unpaired *t* test.

1.3 Supplementary Figure 3

Matched with Figure 3. Myeloid cell linages stained for by flow cytometry in lung **(A)** and BALF **(B)** as a percentage of $CD45^+$ cells (N = 3-5). **(C)** Median fluorescence intensity (MFI) of I-A/I-E on cDC2s from whole lung flow cytometry (N = 3-5). **(D)** Relative mRNA expression of *Ifng* (IFNγ) and *Tbx21* (T-bet) normalized to *Ptprc* (CD45) (N = 3-5). **(E)** B cells by lymphoid panel flow cytometry as a percentage of $CD45^+$ cells (N = 3-5). **(F)** Quantification of multiplex immunofluorescence (COMET) data shown in Figure 3E for B cells ($B220^+$) in lung. ***P* < 0.01, **P* < 0.5 by unpaired *t* test.

1.4 Supplementary Figure 4

Matched with Figure 4. **(A)** Go enrichment GSEA showing the top negatively enriched pathways (N = 4). Cellular deconvolution score of RNA-seq data: common lymphoid progenitor (CLP) by xCell **(B)** and macrophage M2 by CIBERSORT **(C)** (N = 4). **(D)** Correlation of *STAT3* expression vs. CLP infiltration in LUAD (adjusted by stage) (N = 515). Data represent mean ± SEM. **P* < 0.5 by unpaired *t* test.

1.5 Supplementary Figure 5

Flow cytometry gating strategies for myeloid **(A)** and lymphoid (**B**) cells. For the lymphoid gating strategy, CD4^{+} IFNγ^{+} cells are shown as an example of cytokine staining; IFNγ, IL-4, IL-17, FoxP3, and PD-1 were measured in the same manner on B cells and CD8 T cells.

2 Supplementary Tables

2.1 Supplemental Table 1. Antibodies and gating logic used in COMET. Phenotypes with markers in red do not express these markers, which are used as a negative gating strategy.

Immune cells	Gating Order	Phenotype cells & subtypes	Antibodies			
**B & NK cells**	1	B cells	B220+			
	2	NK cells	NK1.1+			
**T cells**	1	CD4 T (non-Treg)	CD4+	FoxP3-	PD-1-	
	2	Treg	CD4+	FoxP3+	PD-1-	
	3	Exhausted Treg	CD4+	FoxP3+	PD-1+	
	4	Exhausted CD4T	CD4+	FoxP3-	PD-1+	
	5	CD8 T	CD8+	GzmB-	PD-1-	
	6	Activated CTL	CD8+	GzmB+	PD-1-	
	7	Exhausted CTL	CD8+	GzmB+	PD-1+	
	8	Exhausted CD8T	CD8+	GzmB-	PD-1+	
**Stem-like T cells**	1	CD4 T	CD4+			
	2	Stem-like CD4T	CD4+	TCF1+		
	3	CD8 T	CD8+			
	4	Stem-like CD8T	CD8+	TCF1+		
**DCs & Macs**	1	Interstitial Macrophages	F4/80+	CD11c-	(Ly6C-)	
	2	Alveolar Macrophages	F4/80+	CD11c+		
	3	PMN	F4/80-	Ly6G+		
	4	Cells	F4/80-	Ly6G-	CD11c-	Ly6C-
	5	DC	F4/80-	Ly6G-	CD11c+	CD86-
	6	Activated DC	F4/80-	Ly6G-	CD11c+	CD86+
	8	Mo/M-MDSC	F4/80-	Ly6G-	CD11c-	Ly6C+
**M1 & M2 -like**	1	M0 Macrophages	F4/80+	CD86-	CD163-	

	2	M1-like	F4/80+	CD86+		
	3	M2-like	F4/80+	CD86-	CD163+	

2.2 Supplemental Table 2. Primers used in qRT-PCR.

Gene	Forward	Reverse
***Actb* (β-actin)**	5’GGCTGTATTCCCCTCCATCG3’	5’CCAGTTGGTAACAATGCCATGT3’
***Ptprc* (CD45)**	5’ACCACCAGGTGAATGTCAATTT3’	5’CTTGCTTTCCCTCGGTTCTTT3’
***Ifng* (IFNγ)**	5’GATGCATTCATGAGTATTGCCAAGT3’	5’GTGGACCACTCGGATGAGCTC3’
***Tbx21* (T-bet)**	5’CAACAACCCCTTTGCCAAAG3’	5’TCCCCCAAGCAGTTGACA3’

2.3 Supplemental Table 3. Antibodies used in flow cytometry.

Myeloid Panel			
**Marker**	**Clone**	**Fluorophore**	**Dilution Factor**
**Ghost Dye™ (Viability)**	-	Violet 510	1:150
**CD45.2**	104	RF710	1:100
**CD11b**	M1/70	FITC	1:100
**CD11c**	N418	PE-Cy7	1:100
**Ly6C**	HK1.4	APC	1:100
**Ly6G**	1A8	PE	1:100

**I-A/I-E**	M5/114.15.2	BV711	1:200
**CD206**	C068C2	BV421	1:50
**CD103**	2E7	PE-Dazzle 594	1:100
**Lymphoid Panel**			
**Marker**	**Clone**	**Fluorophore**	**Dilution Factor**
**Ghost Dye™ (Viability)**	-	Violet 510	1:150
**CD45.2**	104	RF710	1:100
**CD3**	17A2	PerCP-Cy5.5	1:100
**CD4**	GK1.5	APC-Cy7	1:200
**CD8a**	53-6.7	PB	1:200
**CD19**	1D3	APC	1:200
**PD-1**	RMP1-30	BV711	1:100
**IFNγ**	XMG1.2	FITC	1:100
**IL-4**	BVD6-24G2	PE-Cy7	1:100
**IL-17A**	TC11-18H10	BV786	1:100
**FoxP3**	FJK-16S	PE	1:100
**Granzyme B**	NGZB	PE-eFluor 610	1:100

Supplementary Table 4. Mouse plasma.

Mouse Number	Treatment	Final concentration of TTI-101 (ng/ml)
nLR 563	Control	Below LLOQ*
nLR 758	Control	Below LLOQ
nLR 767	Control	Below LLOQ
nLR 556	TTI-101	2940
nLR 686	TTI-101	2400
nLR 749	TTI-101	847
nLR 765	TTI-101	1619

***LLOQ = lower limit of quantitation**
