# Supplementary figures and images for: Selective inhibition of canonical STAT3 signaling suppresses K-ras mutant lung tumorigenesis and reinvigorates anti-tumor immunity
Source: Front Immunol. 2025 Apr 28;16:1575181. doi: 10.3389/fimmu.2025.1575181 (PMC12066534; doi:10.3389/fimmu.2025.1575181)

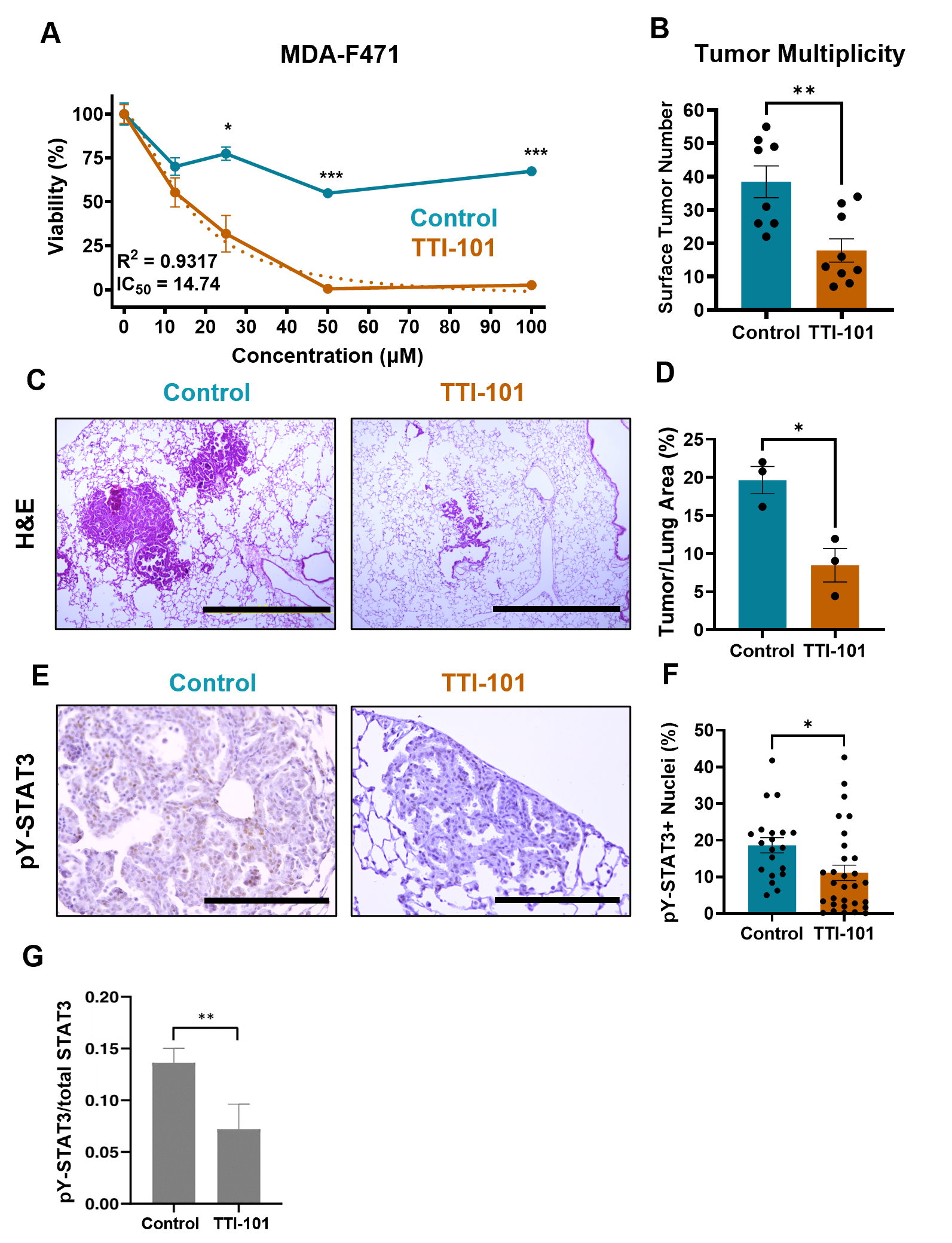

Supplement: Supplementary file 2 [file Image1.tif]

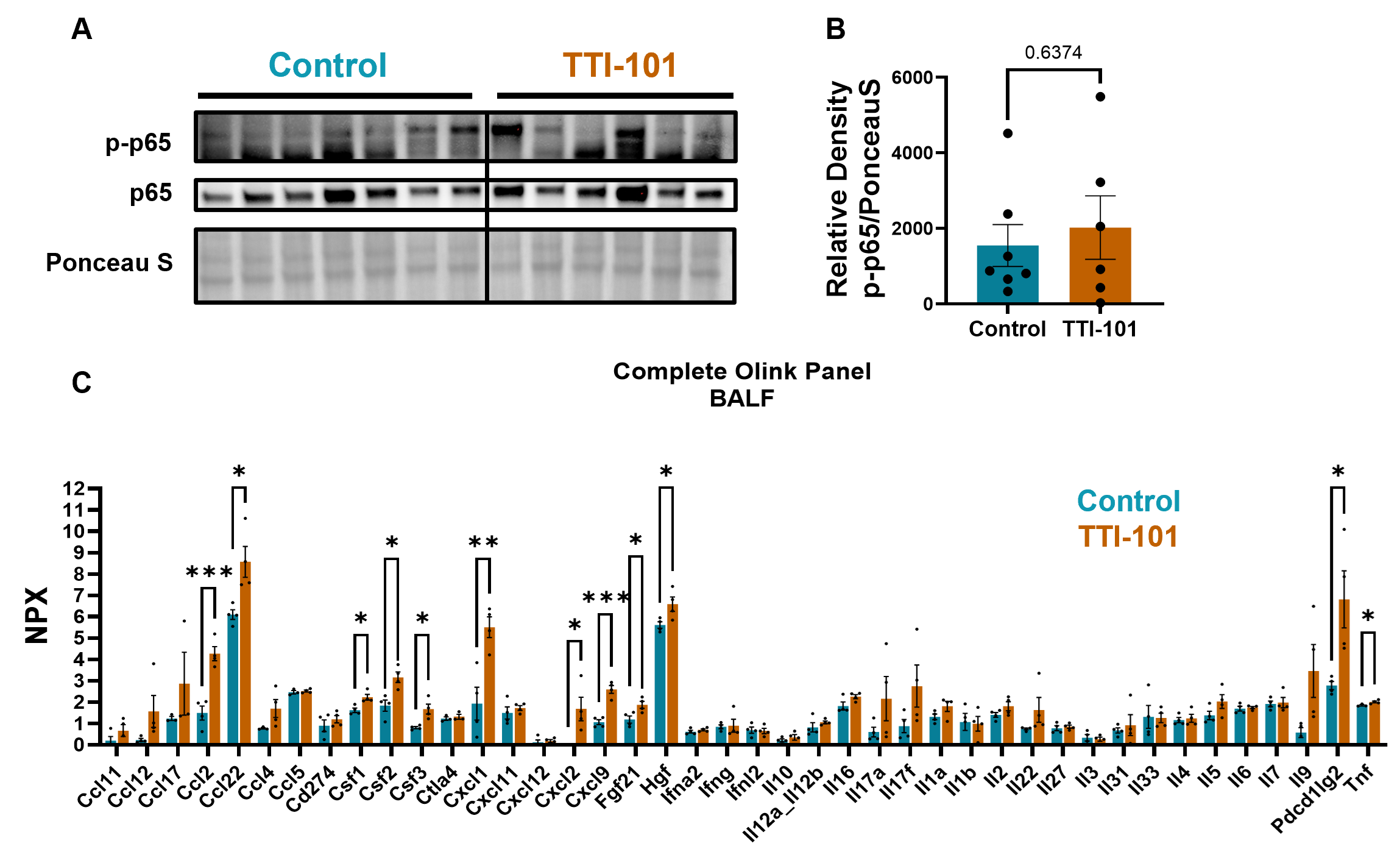

Supplement: Supplementary file 3 [file Image2.tif]

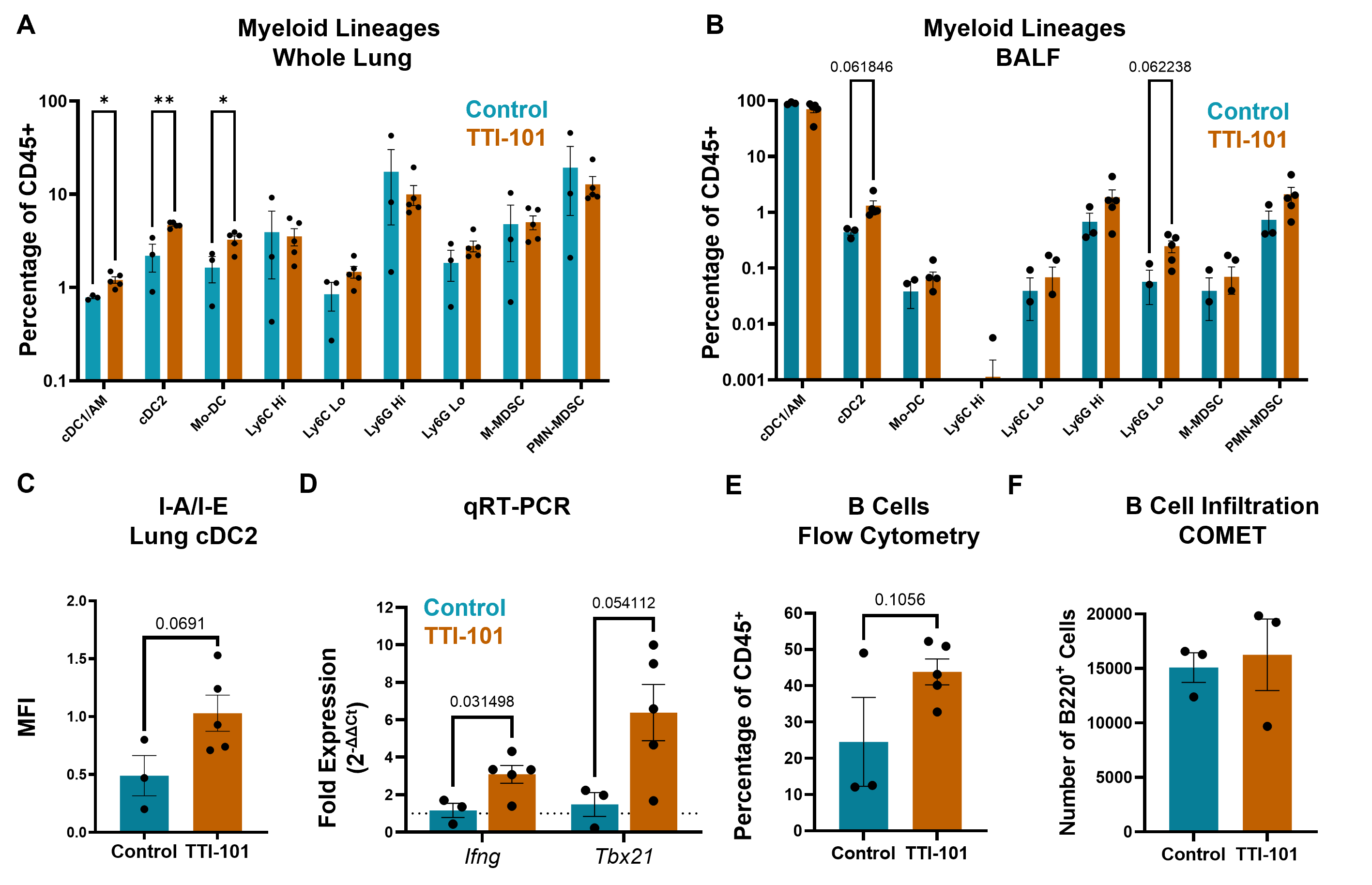

Supplement: Supplementary file 4 [file Image3.tif]

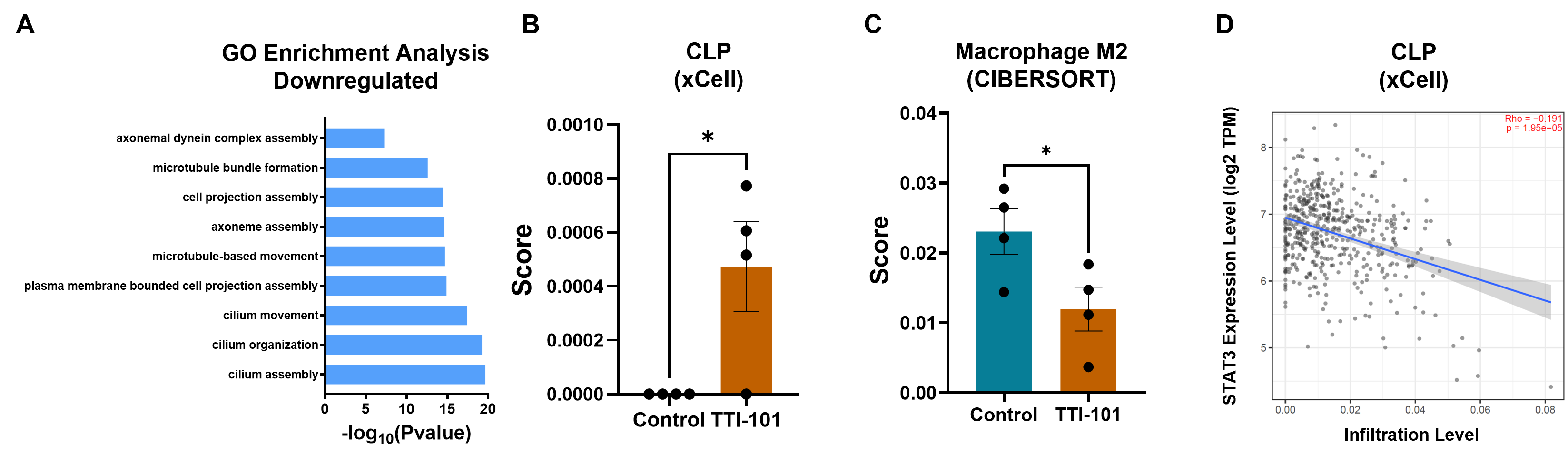

Supplement: Supplementary file 5 [file Image4.tif]

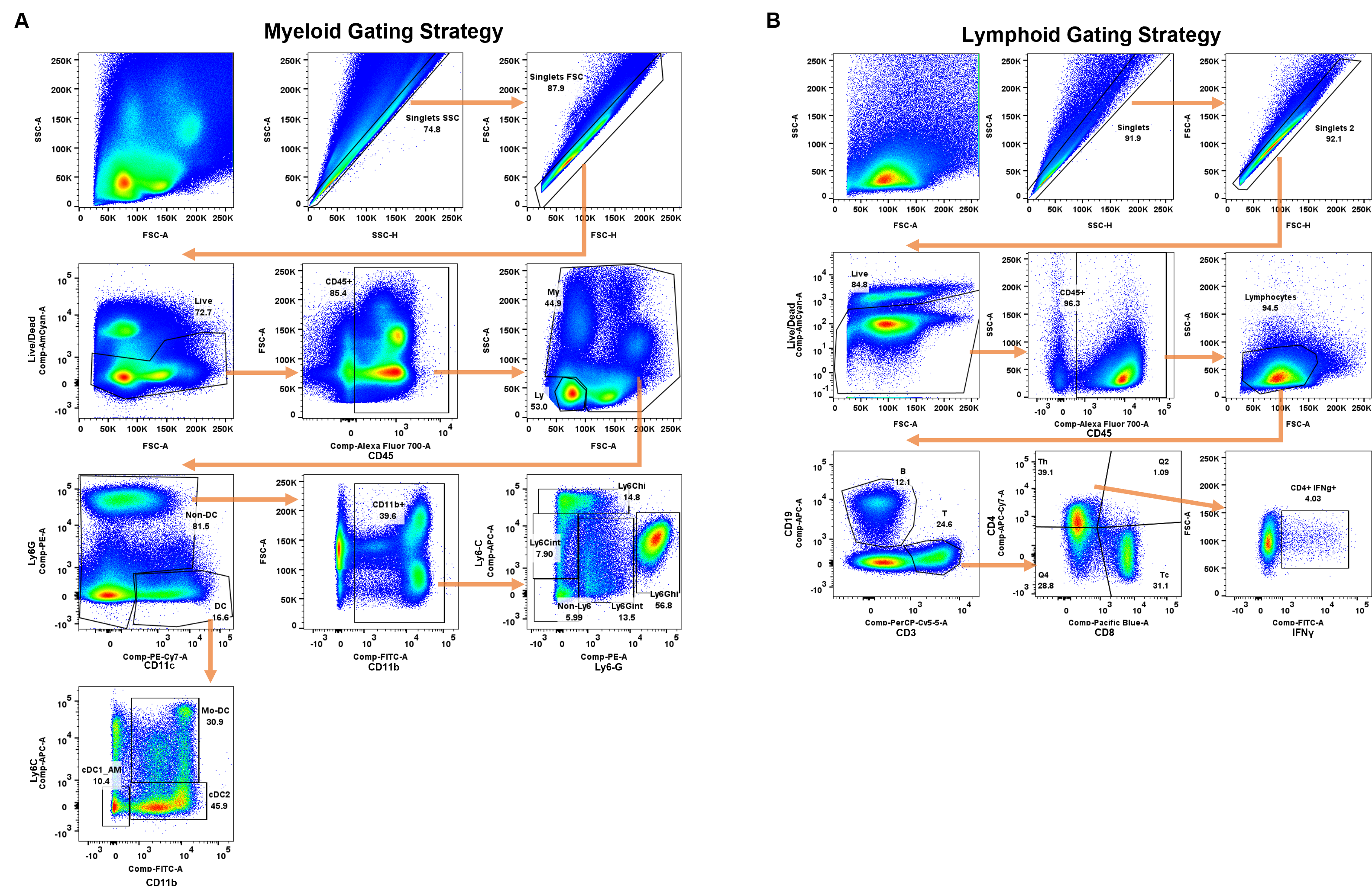

Supplement: Supplementary file 6 [file Image5.tif]
